# The dynamic interplay between acute psychosocial stress, emotion and autobiographical memory

**DOI:** 10.1038/s41598-018-26890-8

**Published:** 2018-06-06

**Authors:** Signy Sheldon, Sonja Chu, Jonas P. Nitschke, Jens C. Pruessner, Jennifer A. Bartz

**Affiliations:** 1 Department of Psychology, McGill University, 2001 McGill College, Montreal, Quebec, H3A 1G1 Canada; 2 Department of Psychology, University of Constance, Universitätsstraße 10, Konstanz, 78464 Germany

## Abstract

Although acute psychosocial stress can impact autobiographical memory retrieval, the nature of this effect is not entirely clear. One reason for this ambiguity is because stress can have opposing effects on the different stages of autobiographical memory retrieval. We addressed this issue by testing how acute stress affects three stages of the autobiographical memory retrieval – accessing, recollecting and reconsolidating a memory. We also investigate the influence of emotion valence on this effect. In a between-subjects design, participants were first exposed to an acute psychosocial stressor or a control task. Next, the participants were shown positive, negative or neutral retrieval cues and asked to access and describe autobiographical memories. After a three to four day delay, participants returned for a second session in which they described these autobiographical memories. During initial retrieval, stressed participants were slower to access memories than were control participants; moreover, cortisol levels were positively associated with response times to access positively-cued memories. There were no effects of stress on the amount of details used to describe memories during initial retrieval, but stress did influence memory detail during session two. During session two, stressed participants recovered significantly more details, particularly emotional ones, from the remembered events than control participants. Our results indicate that the presence of stress impairs the ability to access consolidated autobiographical memories; moreover, although stress has no effect on memory recollection, stress alters how recollected experiences are reconsolidated back into memory traces.

## Introduction

One of the most intriguing characteristics of autobiographical memories - past personal experiences- is that they are not stored and recalled as transcriptions of our past, but rather are accessed and flexibly constructed in our minds as they are retrieved^1–3^. It is generally accepted that episodic memory processes supported by the hippocampus are responsible for the ability to flexibly re-construct autobiographical events^3^ and evidence that episodic memory processes are altered by the presence of acute stress^4,5^ raise questions about how stress modifies the ways autobiographical events are re-constructed. The answers to these questions are likely complex given that autobiographical memory retrieval involves multiple processing stages - from accessing a memory, to dynamically constructing an experience of the event in mind, and finally, reconsolidating it back into a memory representation - that are all affected by stress. Further adding to this complexity, emotion is thought to affect how stress impacts processes relevant to autobiographical memory retrieval^6^. Here, we aimed investigate how stress affects these different stages of autobiographical memory retrieval, and how the effect of stress on autobiographical memory retrieval is influenced by emotion^7–11^.

## The Acute Stress Response and Episodic Memory

The effects of acute stress on episodic memory have been well-researched^4^. A key pattern that has emerged from this line of research is that two variables, namely i) when a stressor occurs and ii) if an emotion is present, moderate the effects of stress on episodic memory. Regarding timing, stress that is experienced during the encoding phase of an episodic memory task is thought to improve performance, particularly if a stressor is applied after learning and during the consolidation phase of memory^12,13^. It is worth noting that the effects of stress on encoding are less consistent if stress is applied prior to encoding, with some studies finding a beneficial effect^14,15^ and others finding a negative effect^16,17^. Stress experienced during the retrieval phase of an episodic memory task is generally thought to impair performance^18,19^.

The opposing effects of stress on encoding and retrieval are explained by models of stress that incorporate two physiological responses^20^. When an acute stressor is experienced, there is a fast-acting sympathetic nervous system (SNS) response that results in the release of catecholamines (e.g., noradrenaline and adrenaline). This is followed by a slower acting system that releases glucocorticoids (e.g., cortisol in humans) via the hypothalamic-pituitary-adrenal (HPA) axis^21,22^. During encoding, noradrenaline and cortisol levels enhance learning by directing memory processes towards the to-be-encoded material [N.B., this effect is enhanced for information that is emotional^23^]. During retrieval, increases in cortisol levels in response to stress impair the efficacy of memory processes supported by brain regions such as the hippocampus and prefrontal cortical structures that are needed for successful remembering^24,25^.

As noted, the effects of stress on encoding and retrieval are also thought to be influenced by emotion^26^. Emotional materials can enhance the presentation of stress effects on memory because brain regions that process emotion (e.g., amygdala) are also a target of the noradrenaline and cortisol stress response^27,28^. Although some studies have found the benefits of stress on encoding and consolidation to be enhanced for emotional material^24,29,30^, a recent meta-analysis reported that post-encoding stress was not consistently affected by the emotional valence of information. This analysis did report, however, that stress during retrieval selectively impairs the access to emotional content^31^.

## Acute Stress and Autobiographical Memory Retrieval

Given the importance of episodic memory in autobiographical memory retrieval, the above-reviewed work would suggest that stress will impair retrieving these experiences, however, findings have been inconsistent^12,23,32^. Early studies using the Autobiographical Memory Test [AMT^33^; found that stress impaired the retrieval of personal past experiences such that fewer specific personal memories were recalled in response to retrieval cues^33,34^; more recent studies, have not replicated this effect^35,36^. Some studies have found that emotion can influence autobiographical memory retrieval, whereas others have not found evidence for this effect^36–38^.

One possible explanation for these inconsistencies is that these reports have not considered that there are distinct stages of autobiographical memory retrieval^39^. Autobiographical remembering begins when there is something in our environment that cues the access of a past personal experience. Once a memory is accessed, there are other processes that will support recovering the details of that memory to help build a representation of it in the mind (recollection). Finally, after a past event is recollected, often it must be re-consolidated back into a memory. A Stress could influence one or all of these stages; moreover, it is possible that stress could differentially influence these processing stages. Here we focus on the effects of stress to the access and reconsolidation stages.

## Autobiographical Memory Access

Memory accessibility is effectively measured by the speed (i.e., reaction time) to generate a memory in response to a cue. Previous work has found that individuals are faster to respond to retrieval cues when memories are accessed directly (i.e., they simply ‘come to mind’) compared to when the memories are more effortful to generate^40–43^. Response time differences in memory retrieval can also measure the way factors like emotion and individual variability in stress responses affect the underlying processes related to memory accessibility^44,45^. Following upon evidence that stressful arousal impedes memory retrieval by limiting cognitive resources^24^, one prediction is that stress will result in slower response times to access memories, due to an increased effort needed to access past events. This also follows an ease-of-retrieval account of memory, which argues that memories can be accessed more directly when there is a match between one’s current retrieval context and the to-be-accessed event^46^. If psychosocial stress can induce a negative state^47^, then a stress-induced negative state would make accessing memories that are of a different emotional valence (i.e., positive) more effortful (i.e., associated with slower response times) than accessing memories that are of same valence (i.e., negative). This prediction is also supported by the mood-memory dependence literature [for a review, see^48^, which assumes that a stress-induced negative state would enhance processing of negatively-valenced information^47^.

## Autobiographical Memory Reconsolidation

Memory reconsolidation occurs when the underlying trace of a retrieved event enters a fragile state and needs to be re-stored into a memory^49–54^. When in this fragile state, that memory trace can become susceptible to alterations based on current retrieval circumstances, which cause that trace to be re-encoded or reconsolidated differently. There are indications that stress may lead to stronger or more detailed memory traces upon updating or reconsolidation. This is because the way stress enhances memory encoding mechanisms, particularly for emotional content^20,55–57^, is similar to how stress can influence memory updating or reconsolidation mechanisms^50,58^. That is, if memory retrieval is thought of as a secondary encoding opportunity, then retrieving memories under stress will lead to recollections that are more strongly reconsolidated back into memories^59,60^. Moreover, based on the notion that the encoding enhancement of stress is most robust for emotional content, this proposed reconsolidation enhancement should be most robust for emotional material^16,61^. Testing this hypothesis in humans with autobiographical memories is important, mostly because the majority of the literature supporting this finding stems from animal models (for a review, see^62^), with only some human studies reporting the detrimental effects of stress on reconsolidation^37,63^.

## The Current Study

**Figure 1:**
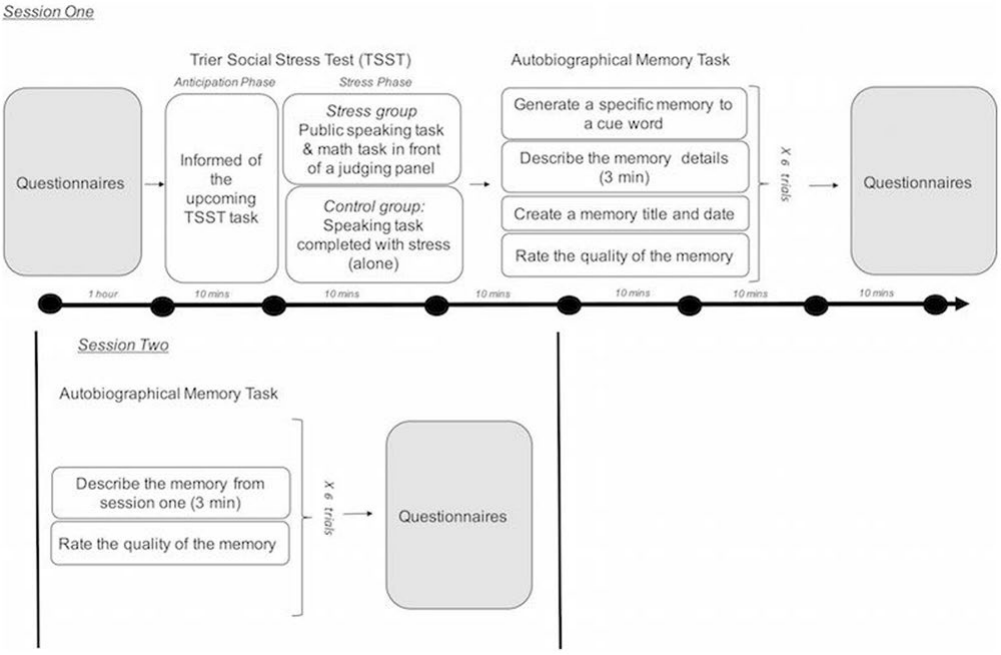
A schematic of the experimental procedure used in the current study.

Summarizing above, we tested two hypotheses about the impairing and enhancing effects of stress on 1) accessing autobiographical memories and 2) reconsolidating the associated recollections. We did this by pairing a widely used and reliable psychological stress protocol, the Trier Social Stress Test TSST^17,64,65^, which experimentally elicits the psychological and physiological stress response in laboratory settings, with a well-validated measure of detailed autobiographical memory retrieval (Fig. 1). Critically, our autobiographical memory task involved two testing sessions separated by a long delay (72 to 96 hours) so that we could measure processes that support memory retrieval, recollection and reconsolidation. We had two primary predictions. First, during initial retrieval, we predicted that stress would impair the ability to access autobiographical memories, and that this effect would be linked to the emotional content of a retrieval cue and the cortisol stress response. That is, following the aforementioned matching hypothesis, we predicted that stress would impair retrieval for positive but not negative memories. Second, regarding the effects of stress on recollection and reconsolidation, if retrieval serves as a secondary encoding context, we predicted that the memories initially retrieved under stress will be better recalled during the subsequent testing session and this effect will be most robust for the emotional content of the memories.

## Methods

### Participants

Forty-eight young, healthy, male, university student volunteers between the ages of 18 and 30 years old were tested. We restricted our sample to male participants because HPA reactivity varies considerably in females due to hormonal fluctuations throughout the menstrual cycle^66^ and, for this reason, it is necessary to carefully track menstrual cycle phase when having female participants undergo the TSST. Because this is the first study of its kind, we sought to establish the basic effect in the more homogenous male sample to serve as proof of concept for future research. All the tested participants were fluent in English and were free from factors that could affect stress reactivity (i.e., no prior knowledge of the TSST, consumed on average fewer than 10 units of alcohol and tobacco per week, no illicit drug use, did not endorse symptoms associated with depression and/or anxiety). In addition, participants were unaware that the present study was about the effects of stress on memory and were recruited for a study on personality. We excluded four participants from the analysis, two for not meeting the above-criteria, one participant withdrew from the study and one was excluded for incomplete data collection. Thus, the final sample consisted of 44 participants (mean age = 22 years, SD = 2.6, range 18 to 30 years). All participants provided informed consent prior to the study and were compensated monetarily for their time. The study was approved by the McGill University Faculty of Science Institutional Review Board and the experiment was conducted in accordance with the associated guidelines and regulations.

### Experimental design

Participants completed two experimental sessions that took place three or four days apart. Each session occurred in the same testing room and at the same time, which was always between 1 pm and 6 pm because of known diurnal changes in stress hormones like cortisol. All participants were instructed not to drink alcohol the night before and refrain from eating or drinking two hours before the start of the first session to ensure accurate measures of stress reactivity (cortisol and salivary alpha-amylase (sAA)) from collected salivary samples. During session one, half of the participants were randomly assigned to the stress group and half to the control group. As part of a larger testing battery, the experimental procedure for session one started with a series of questionnaires and computerized tasks. These were followed by the stress protocol and the session ended with an autobiographical memory test as well as other questionnaires and computerized tasks. During session two (3 or 4 days later), participants were administered the same autobiographical memory test. The tasks are described in detail in the following sections and readers can refer to Fig. 1 for a visual depiction of the experimental paradigm. The datasets generated during the current study are available from the corresponding author on request.

### Session one: Stress protocol

For both groups, session one began with a series of questionnaires and an unrelated experimental task that lasted approximately one hour to reduce any anticipatory or pre-experimental stress levels. The participants in the stress group then completed the TSST^17^, which involved presenting an impromptu speech and doing a challenging arithmetic task in front of two confederates posing as “expert” judges (trained confederates, one male and one female). Specifically, it constituted a mock-job interview in which the participant was given 10 minutes to prepare a speech to be given in front of the two judges who remained expressionless during the task. Following this anticipation period, participants performed a 5-minute speech, followed by a 5-minute arithmetic task in front of both judges. The TSST has been shown to reliably produce a significant increase in stress at all levels: cortisol, sAA, and subjective stress^67–69^. The control group performed a closely matched non-stress inducing task^70^. This task involved standing in a room alone (i.e., without any of the stressful social or evaluative components of the TSST) and talking out loud about a movie, book or vacation and then performing an easy arithmetic addition task.

For both groups, cortisol and sAA levels were collected via salivary samples to measure stress reactivity throughout this session. For each saliva sample, participants inserted a cotton swab (‘Salivette’; Sarstedt, Saint-Léonard, QC) inside their mouth without touching their lips or fingers and chewed on the swab for one minute. Cortisol levels (nmol/l) were measured using a time-resolved fluorescence immunoassay described by Dressendörfer, *et al*.^71^. sAA (U/ml) levels, which measure the SNS response, were determined using the enzyme kinetic method referred to in Engert, *et al*.^65^. Subjective stress was also evaluated using visual analogue scales (VAS) at each saliva sampling. Ten rating measures were collected by participants marking an ‘x’ on a 11 cm line with two anchors (0 = “not at all”, and 10 = “very much”) to indicate how they felt in that moment. For this study, we only focused on the ‘How stressed do you feel right now?’ scale. All measures were anchored to 7–8 time-points, in 10-min intervals, throughout the experiment from −20 to +60 min.

### Session one: Autobiographical memory task

Immediately following the stress protocol, participants were given an autobiographical memory task. On a computer screen, participants were randomly presented with six cue words of different valence taken from previously published studies^72^. This included two positive words (happy, interesting), two negative words (sad, angry), and two neutral words (busy, concentrated). To each cue word, the participants were instructed to recall an associated specific past personal event as quickly as possible within two minutes. They were told that the event should be one that happened to them, happened in one location and happened over minutes or hours and no more than one day. To control for the age of the retrieved memories, participants were instructed to only choose memories that occurred in the past 6 months. When they accessed an event, they pressed ‘1’ on the keyboard as soon as possible and their reaction time was measured. The participants were then given up to three minutes to describe the accessed event and were told to describe out loud as many event details as possible. These descriptions were recorded and transcribed for later scoring. Each trial ended with participants generating an event title (a short phrase that summarized their memory that was to be used as a memory cue in session two), estimating the date the event occurred, and rating the quality of the remembered event on five measures (Table 1). Given that each event recall and description could take up to five minutes, session one’s memory task lasted a maximum of 30 minutes.

**Table 1:** Subjective rating measurements collected to each specific event described for the autobiographical memory task.

Rating Measure	Participant Prompt
Emotional Valence	What was the emotion of the event? (1 - negative, 6 - positive)
Emotional Intensity	How strong was that emotion? (1- very weak, 6 -extremely strong)
Vividness	How vividly can you picture this event in your mind? (1 – vague, 6 – a lot of detail)
Importance	How important is this event to your sense of self? (1 – not at all, 6 – extremely)
Rehearsal	On average, how often do you think or talk about this event (1 – first time, 6 – daily)
Intrusions*	Since the last experiment, how much have you thought about this memory? (1 – first time, 6 – quite a bit)
Change*	Since the last experiment, do you think your memory for this event has changed? (1 – not at all, 6 – a lot)

*Assessed in Session 2.

### Session two: Autobiographical memory task

Either three or four days later (variability due to scheduling issues), participants returned to the same laboratory testing room and were randomly presented with the event titles they generated during the first session on a computer screen. To each cue, they described out loud in three minutes as many event details as possible, as they did in session one. These descriptions were recorded and transcribed for later scoring. Participants then made the same ratings as in session one as well as two additional ratings (see Table 1).

### Autobiographical memory scoring: Event type and details

Each described event was categorized using the scoring protocol associated with the AMT^33^. This protocol classifies events as either a specific memory (An event that lasted less than a day; e.g., “dinner party at Alan’s house last summer”) or a non-specific memory (extended memory - An event that lasts more than a day and is not specific to a single spatial context; e.g., “A road trip to Toronto”; categorical memory - an event that occurs repeatedly over time; e.g., “Going to spin class in the morning”; semantic associate - a response that contained factual information; e.g., “I am a happy person”).

The transcribed autobiographical memory descriptions were scored with the procedure of the Autobiographical Interview^73^. This protocol segments and quantifies components of narratives into details, defined as “a unique occurrence, observation, fact, statement, or thought…that independently conveys information”, which are then categorized as internal or external details. Internal details reflect the extent of episodic recollection for a specific memory as these details pertain directly to the defined event and its spatial and temporal contexts. These internal details are further categorized as event, place, time, perceptual, or emotion/thought details. In this study, we focused on the number of emotion/thought details, which are defined as descriptions of one’s own emotional state at the time of the event. External details provide information about the described memory that is not directly related to the main event described and include details regarding a tangential event, semantic information, or metacognitive statements expressed at the time of testing.

### Autobiographical memory scoring: Affect rating

To obtain an independent rating of the emotional quality of the descriptions, we used the Linguistic Inquiry Word Count (LIWC), which is a text analysis program that compares each word in a document (in this case, the memory descriptions) to a large corpora of dictionaries and catalogs to classify them into a variety of categories (e.g., past orientation, emotional tone). To test our specific hypotheses, we focused on the number of words for each description of the memories that were classified as affective, or containing emotional content.

### Data analysis

We first ran a mixed measures analyses of variance (ANOVA) on the collected emotional valence ratings for each of the memories generated during session one as a function of cue type to confirm that cue valence was reflected in the autobiographical memories and to justify including cue type as a within-subject factor in subsequent analyses. For this analysis, valence ratings were coded as either positive (ratings of 4, 5, and 6) or negative (ratings of 1, 2, and 3). Then, we calculated the proportion of memories that were rated as negative for each cue type condition and entered this as the dependent variable.

To test predictions about how stress affects memory access, we ran a 2 (group: stress vs. control) x 3 (cue-type: positive, negative, vs. neutral) repeated measures ANOVA on the average response time to recall an autobiographical memory during session one. Prior to analysis, we visually inspected the response time distribution, which revealed a non-normal distribution so these response times were log-transformed. Three identified outliers (over 3 SD above the mean) were excluded from this analysis.

We examined how stress affected memory recollection during session one by averaging the number of internal and external details generated in the descriptions of recalled memories and running a 2 (group: stress vs. control) x 3 (cue-type: positive, negative, vs. neutral) x 2 (detail: internal and external) ANOVA. We examined how stress affected memory reconsolidation with a similar ANOVA using data from session two. Significant group differences from these analyses were followed with ANOVAs with the number of emotional details contained in the memory descriptions as the dependent variable that were then followed by an ANOVA on the affective content of the memories as determined by the LIWC.

Finally, we explored significant effects of stress related to the physiological stress responses (cortisol and sAA) using correlation analyses. We used Pearson’s and Spearman’s correlation for continuous or ordinal variables, respectively.

## Results

### Group demographics

Table 2 illustrates the characteristics of the participants in the stress and control (placebo TSST) group. Of note, there were no differences in mean age or depression scores (BDI-II^74^), nor were there differences in baseline mood at the time of testing (PANAS-20^75^). Confirming our experimental manipulation of stress, the groups differed in measures of stress reactivity reported here as area under the curve (AUCi) measures – computed using the trapezoid formula described by Pruessner, *et al*.^76^. To measure the effects of stress on changes in mood we computed the delta-peak – absolute changes from baseline to peak stress for the VAS measure.

**Table 2:** Stress response in the stress and control group.

	Stress	Control	F	p
Age (years)	21 (0.5)	22 (0.7)	0.61	0.44
Handedness	17 (R)	15 (R)		
BDI-II scores	31.4 (1.8)	30.8 (1.9)	0.20	0.83
*Baseline Measures*:				
PANAS positive	31.01 (1.73)	30.95 (2.02)	0.02	0.90
PANAS negative	15.54 (0.94)	18.35 (1.37)	1.77	0.20
*Stress Response*:				
Cortisol AUCi levels (z scores)	27.41 (5.96)	−1.30 (1.43)	20.51	<0.001***
Amylase AUCi levels (z scores)	17.23 (4.14)	6.23 (2.62)	5.18	0.03*
Delta-peak VAS	3.40 (0.51)	0.10 (0.13)	37.11	<0.001***

Levels of significance: 0 ‘***’ 0.001 ‘**’ 0.01 ‘*’ 0.05 ‘.’ 0.1.

### Session One: Effects of Stress on Retrieval

#### Memory valence

To confirm that cue type categorization was reflected in the autobiographical memories, we ran a mixed ANOVA on the emotional valence ratings. There was a main effect of cue-type (*F*(1,42) = 45.45, *p* < 0.001, partial η² = 0.51) as well as group (*F*(1,42) = 5.95, *p* = 0.02, partial η² = 0.13). As expected, there was a stepwise increase in the proportion of memories that were negative, increasing from the positive to neutral to negative cue condition. Interestingly, the group effect was due to a higher proportion of memories rated as negative for the stress (0.52, SE = 0.04) compared to the control group (0.40, SE = 0.04).

#### Reaction time

When we examined differences in the response time to generate autobiographical experiences as a function of group (stress vs. control) and cue type (positive, negative and neutral), the results showed a main effect of group (*F*(1,40) = 4.31, *p* = 0.04, partial η² = 0.10) but no effect of cue-type (*F*(2,80) = 0.35, *p* > 0.25, partial η² = 0.008) nor an interaction effect (*F*(2,80) = 1.53, *p* > 0.25, partial η² < 0.04). Irrespective of cue type, participants in the stress group were slower to access specific autobiographical events (mean = 35 seconds; SE = 2.8 seconds) than those in the control group (mean = 28 seconds; SE = 2.7 seconds). Critically, there were no group differences in the average number of specific memories generated in response to these cues (group (*F*(1,43) = 0.71, *p* > 0.25, partial η² = 0.02), cue-type (*F*(2,86) = 2.37, *p* = 0.10, partial η² = 0.05), or an interaction effect (*F*(2,86) = 0.81, *p* > 0.25, partial η² = 0.02), indicating that the slower response times in the stress group were not due to this group generating a different ‘end-product.’ This group difference also held when we examined reaction times to generate only specific memories.

**Figure 2:**
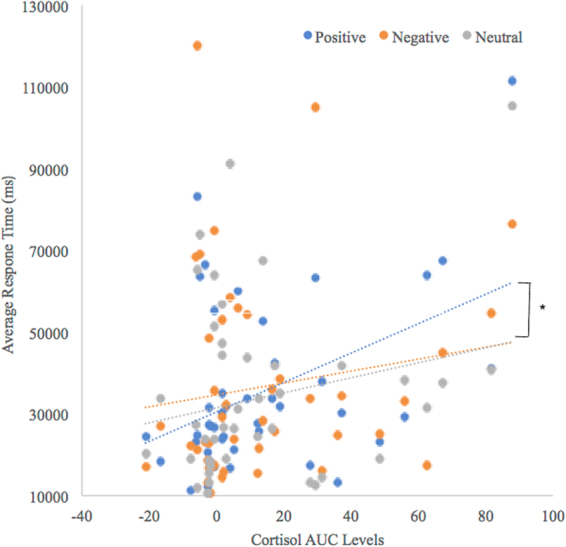
The correlation between the cortisol response (area under the curve levels) and the average participant response time to generate memories as a function of cue word valence. The correlation between the cortisol levels and response times was significantly greater for positively- cued memories compared to negatively- and neutrally-cued memories.

To determine which stress hormone was related to memory access performance, we correlated cortisol AUCi levels to these response times with a series of Pearson correlation analyses. Here, we found that cortisol levels positively correlated with response times to access memories to positive cue words (*r* = 0.44, *p* = 0.003), but was not significantly correlated with time to access negative (*r* = 0.15, *p* = 0.32), or neutral cues words (*r = *0.21, *p* = 0.17, see Fig. 2). Fisher’s r-to-z transformation was used to test if the correlation values for positive and negative as well as positive and neutral cue words were significantly different from one another after accounting for dependency within the data. The z-score based on the difference between positive and negative cues (Z = 2.20) was significant (p = 0.01) as was the difference between positive and neutral cues (Z = 1.66; p = 0.05). sAA AUCi levels did not correlate with response times for any cue category (positive cues: *r* = 0.02, *p* = 0.92; negative cues: *r* = −0.03, *p* = 0.88; neutral cues: *r* = 0.08, *p = *0.63).

#### Memory details and ratings

The ANOVA run on the average number of details generated when describing memories, with group (stress, control), cue-type (positive, negative and neutral) and detail type (internal, external) as factors, showed no main effects of group (*F*(1,42) = 0.08, *p* > 0.25, partial η² = 0.002), cue-type (*F*(2,84) = 1.00, *p* > 0.25, partial η² = 0.001) or any group interactions between these factors (cue-type and group: *F*(2,84) = 0.68, *p* > 0.25, partial η² = 0.016; detail-type and group: *F*(1, 42) = 0.12, *p* > 0.25, partial η² = 0.003; group, cue-type and detail type, *F*(2,84) = 0.46, *p* > 0.25, partial η² = 0.01), although the interaction between cue-type and detail type did appear to approach conventional levels of statistical significance, *F*(2,84) = 2.75, *p* = 0.07, partial η² = 0.06). The lack of a group effect precluded us from examining the effect of stress on emotional detail generation. We also found no group differences when we examined the ratings (vividness, emotional intensity, rehearsal, and importance) with a M(ultivariate)ANOVA (top panel, Table 3).

**Table 3:** The average ratings for memories recalled during session 1 and 2. Standard error is shown in parentheses.

Session 1	Stress	Control	F	p
Vividness	4.5 (0.17)	4.7 (0.17)	1.16	0.29
Emotional Intensity	4.1 (0.16)	4.4 (0.19)	1.07	0.31
Importance	3.1(0.13)	3.7 (0.13)	3.23	0.08
Rehearsal	2.2 (0.13)	2.4 (0.62)	0.75	0.39
**Session 2**	**Stress**	**Control**	**F**	**p**
Vividness	4.1 (0.21)	4.3 (0.20)	0.46	0.50
Emotional Intensity	3.9 (0.16)	4.0 (0.19)	0.07	0.79
Importance	3.0 (0.21)	3.3 (0.25)	1.07	0.31
Rehearsal	2.3 (0.14)	2.5 (13)	0.70	0.41

### Session Two: Effects of Stress on Reconsolidation

#### Memory details and ratings

**Figure 3:**
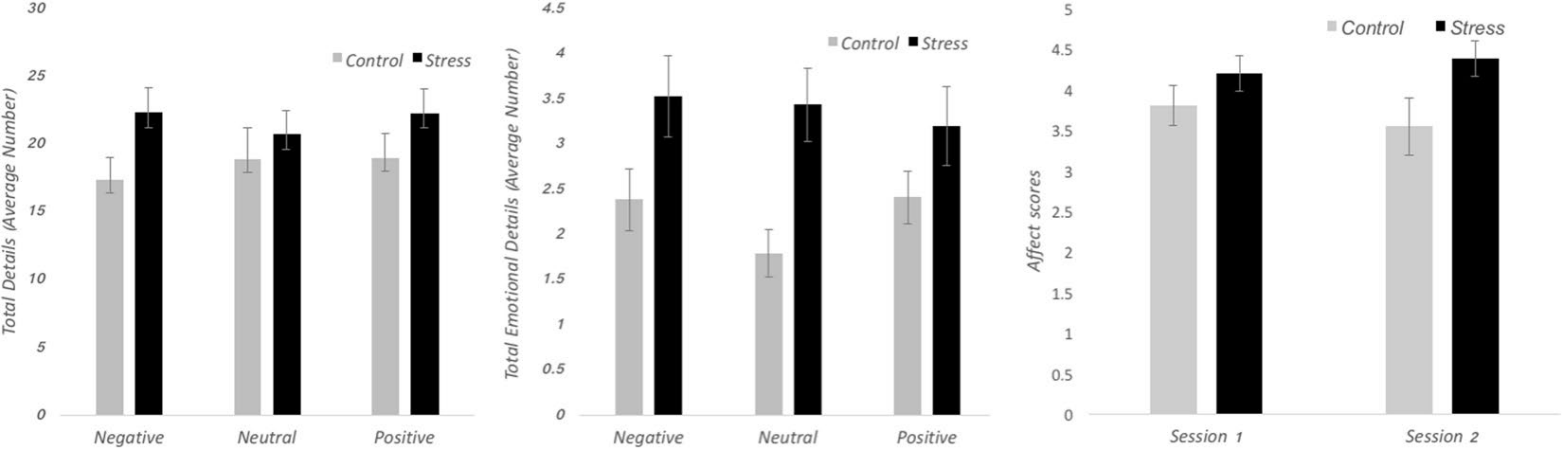
Left panel: The average number of total details generated when recalling autobiographical memories during session two. There was a main effect of group such that the stress group generated more details than the control group and here the average details are presented as function of word cue type for illustrative purposes (i.e., the interaction between group and cue type was not significant). Middle panel: The average number of emotional details generated when recalling autobiographical memories during session two. Overall, the stress group generated more emotional details than the control group. As in the left panel, the averages are presented as a function of word cue type for the stress and control group for illustrative purposes (i.e., the interaction between group and cue type was not significant). Right panel: The average affect ratings from the LIWC for the autobiographical memories recalled during session one and two for the stress and control group. For all figures, the error bars shown represent standard errors.

The mixed design ANOVA with condition, cue type, and detail type as factors with the number of total details generated during session one included as a covariate resulted in a main effect of group, (*F*(1,41) = 4.47, *p* = 0.04, partial η² = 0.10), but no effect of cue-type and group (*F*(2,82) = 0.33, *p* > 0.25, partial η² = 0.008) nor between group and detail type (*F*(1,41) = 0.001. *p* > 0.25, partial η² < 0.001). Those in the stress group remembered overall more details from the re-retrieved memories compared to the control group (Fig. 3, left panel, depicts group differences by cue type for illustrative purposes – only the main effect of group was significant). There were no group differences when the ratings (vividness, emotional intensity, rehearsal, and importance) were compared (bottom panel, Table 3).

The significant group effect on detail generation was followed by a mixed-design ANOVA with group and cue-type as factors on the number of emotional details remembered. The total number of internal details generated during session one was included as a covariate. As expected, there was a strong effect of group (*F*(1,42) = 7.28, *p* = 0.01, partial η² = 0.30) and no other significant effects. Illustrated in Fig. 3 (middle panel), those in the stress group recalled more emotional details at session two, irrespective of cue-type.

We ran two analyses to follow up on the link between stress and emotional detail recall. First, we assessed the link between the increase in emotional detail recovery and initial stress hormone levels with a series of correlations (n.b., Spearman’s correlations were used here because details are an ordinal variable) between cortisol and sAA AUCi measures from session one and the number of emotional details generated during session two for each cue type. Initial (i.e., session one) cortisol levels significantly correlated with generating emotional details to neutrally-cued memories during session two (positive cues: *r* = 0.19, *p* = 0.23; negative cues: *r* = −0.08, *p* = 0.64; neutral cues: *r* = 0.37, *p = *0.02). Initial sAA AUCi levels also correlated positively with the number of emotional details generated to neutral cues during session two (positive cues: *r* = 0.27, *p* = 0.08; negative cues: *r* = 0.25, *p* = 0.11; neutral cues: *r* = 0.39, *p = *0.009). Fisher’s r-to-z transformation test indicated the correlation between sAA and cortisol levels to neutrally-cued memory’s emotional detail count was not significantly different (Z = 0.80; p = 0.21).

Second, we confirmed the link between emotion detail recovery and stress with the LIWC text analysis software that provided an overall emotional (affect) content rating of the memory descriptions, which we could compare between the groups. For this analysis, one outlier participant’s data was excluded for being three standard deviations above the mean affect rating score from the LIWC. An ANOVA with group and cue type on the affect scores revealed a main effect of cue type (*F*(2,80) = 5.14, *p* = 0.008, partial η² = 0.11), no interaction between cue type and group (*F*(2,80) = 1.49, *p* = 0.23, partial η² = 0.04), and critically, a main effect of group, (*F*(1,40) = 4.24, *p* = 0.046, partial η² = 0.04). Memories described by the stress group were significantly more emotional (i.e., higher affect rating) than those described by the control group during session two (Fig. 3, right panel). When we ran this analysis with the descriptions from session one, this group difference was not present (*F*(1,40) = 1.38, *p* = 0.25, partial η² = 0.03; Fig. 3, left panel), suggesting the group effect on affect ratings was selective to session two.

## Discussion

A common finding is that stress negatively affects episodic memory retrieval^77^. This finding has not been consistent for studies examining the use of episodic memory during autobiographical remembering. The fact that autobiographical memory retrieval involves many different stages of retrieval^78^ may be a reason for these mixed findings^6^. To address this issue, our study characterized how acute psychosocial stress affected different stages of autobiographical memory retrieval: accessing, recollecting, and reconsolidating a remembered experience. Our results showed a trade-off in how stress affected accessing and reconsolidating these personal long-term memories. First, acute stress impaired the ability to access autobiographical memories in response to retrieval cues, as indicated by a slower response time to recall past personal events in stressed as compared to non-stressed individuals. Second, acute stress did not affect how these accessed memories were immediately recollected (i.e., the amount of detail generated when describing the memories under stress), but it did strengthen the ability to later recover specific details– in particular, the emotional details of these recollected memories - 72 to 96 hours later. Below we discuss the possible mechanisms and adaptive functions of these two seemingly opposite effects of stress on different stages of autobiographical memory retrieval.

First, our finding that stress increases the time to access autobiographical memories to a retrieval cue suggests that stress makes accessing consolidated personal memories less direct and more effortful. This notion is based on reports that fast response times to memory retrieval cues indicate taking a direct route to recalling a past experience (i.e., the memory simply comes to mind) whereas slow response times to a memory retrieval cues represent the use of more generative or effortful memory processes^41^.

There may be some adaptive functions for limiting access to the resources used to recall past memories when under stress. When faced with a stressor, one could imagine that it would be more adaptive to direct one’s mental resources towards *encoding* the current environment – the source of stress – and away from  accessing past memories that may not be relevant to one’s current scenario^79^. As such, it is likely that certain memories may be most prone to retrieval failure under stress than others. In particular, if stress is viewed as a negative state, memories that are emotionally incongruent with this state (i.e., positive) should be the ones most difficult to generate/access. This hypothesis is based on mood-congruency findings indicating that emotional material is more readily accessed (less effortful) when it matches a current mood state^80^. Although we did not find a group difference in response times as a function of memory cue valence, we did find that elevated cortisol levels were selectively related to more effortful processing of positive memory cues, consistent with the mood-congruency theory. Interestingly, cortisol but not sAA levels, which measure the fast acting SNS response, were related to response times. In fact, cortisol levels have been linked to effortful retrieval tasks^4^, which would explain why we found this link -  a greater cortisol response to stress interfered with accessing positively cued memories since these types of memories are the most effortful to access. Even though we find stress hampered the ability to access past memories, there could be some stressful scenarios that would make personal memories adaptive to retrieve. For example, recalling memories that are relevant to the current stressful situation would allow one to identify methods used in the past to cope with the stressor. Although we did not find direct support for this idea, we did find that the stress group was more likely to access memories that were rated negative in valence than did the control group - an emotional valence that matched their current situation if one assumes that stress induces a negative emotional state^47^.

While the response time to generate specific memories differed between the stress and no stress groups, the number of specific memories recalled did not differ between groups. Although fitting with some prior work^81^, this result differs from other investigations showing that stress impairs the ability to generate specific memories^34,38^. Methodological differences may account for these mixed results. Many studies investigating the effects of stress on accessing specific autobiographical memories do not require participants to describe memories in detail, but ask for a brief (i.e., one sentence) label of the accessed memory. By contrast, our participants knew they would be asked to describe the specific details of the recalled events, which may have modified how they initially ‘captioned’ or labeled their retrieved memories. Assessing detailed descriptions of recalled memories is a more sensitive marker of how autobiographical memories are retrieved (i.e., recollected) than scoring the type of event recovered^73,82–85^, yet even with this marker, autobiographical memories were recollected similarly by the two groups during the first testing session. As we predicted, however, memories initially recollected under stress were retrieved with more details, particularly emotional details, when retrieved after a delay. Stress affected the reconsolidation of these long-term consolidated autobiographical memories.

Specifcally, we found evidence that although stress impaired initial access to a memory, it seemed to enhance some aspects of the memory trace via reconsolidation or updating. Some have argued that reconsolidation can be viewed as a secondary encoding opportunity that is similarly sensitive to factors that affect memory formation^9,11^. If stress enhances memory encoding, this would explain how the presence of stress strengthened memory recollection during session two - it enhanced the re-encoding of the recollected details into the underlying memory trace when this trace was destabilized during the initial testing session. Of note, this reconsolidation effect of stress was particularly strong for the emotional content of the autobiographical memories^61^. In fact, when we correlated the initial (session one) physiological stress responses to the number of emotional details recalled during session two, there was a positive correlation between glucocorticoid (cortisol) and catecholamine (sAA) levels and the amount of emotional details that were later recovered for neutrally-cued memories. This finding extends work showing that both of these physiological stress responses (glucocorticoid and catecholamine) enhance encoding mechanisms^28^ to reconsolidation mechanisms  by indicating that these levels allow details of a recalled event to be re-encoded more strongly^23,27,86,87^. Interestingly, this pattern specifies that these levels relate to the reconsolidation of the emotional details of an event when that event is not, in and of itself, emotional (i.e., neutrally cued).

The fact that an association between the physiological stress responses and the emotional detail recovery was not present for all the cued memories raises questions about what other mechanisms may underlie the reported reconsolidation effect. One possibility is that this is the result of a mood-congruency effect induced by the stressor. We administered the stressor prior to the autobiographical memory test, which may have induced a negative mood state in the participants. While this possible state did not affect the details initially recalled when describing memories, it may have heightened attention to or monitoring of the recalled emotional details, which resulted in a preference to re-encode the emotional materials present in a consolidated memory trace^88^.

Alternatively, stress during retrieval may heighten emotional arousal, and consequently, lead to the incorporation of new retrieval-state-based emotional content into the memory trace^50^. In our study, participants recalled autobiographical memories during session one and two in the same experimental room. Since context serves as a strong reminder of past experiences, this manipulation may have biased participants to recover the autobiographical memories from session one’s remembering experience - which could have led the stress group to also recall the arousal they felt at that time.

Despite finding an effect of stress on reconsolidating the emotional content of retrieved autobiographical memories, there are findings inconsistent with this result (^59,60,89^; for a review on animal findings^62^). For example, one study found that externally administering cortisol as participants retrieved previously studied wordlists impaired the ability to recall these words after a week long delay^37^. Yet, a reason for the difference outcomes between this study (also see^90^) and ours is the memories that were being evaluated. While this study used wordlists, we tested complex remote autobiographical events that require several interacting reconstructive processes for successful retrieval. Autobiographical memories and stimuli like wordlists are recalled using different neural mechanisms^91^, and thus may be differently affected by stress.

Another major difference to consider between our study and those with different outcomes is how the stressor was administered. In our study, we used a psychosocial stressor to induce a cortisol stress response, whereas other work has found different effects when cortisol was directly administered^37^. For example, in the study noted above,  Tollenaar *et al*. (2009) administered hydrocortisone. Hydrocortisone administration increases bioavailable cortisol, but the experience of a psychosocial stressor leads to a more complex response, involving not just the HPA axis with its downstream marker cortisol, but also a robust activation of the SNS, as well as increased feelings of psychological distress^64^. Since the appraisal of a situation plays an important role in the cognitive strategies used for a social task^92,93^, the psychosocial nature of the TSST might have influenced the recall of autobiographical memory, which often has a social component to it.

 There are other methodological and analytic issues related to our study worth mentioning. One issue is that the size of the group effect (stress vs control) we report was relatively small when we examined the overall number of details recalled. However, when we focused our analyses on the emotional details generated when describing the memories, the group effect was larger. We were also able to confirm this effect when memories were assessed for emotional language using a text analysis tool - memories initially recalled under stress were later remembered with more emotional words than those not initially recalled under stress. Another issue with our study is that our experiment included only male participants. Research indicates that males and females can differ in their stress responses^94^ and can show differential effects of stress on memory^14,95,96^. An important next step in this line of research is to determine whether the reported pattern of results extends to females. A final issue concerns the timing of our stressor with respect to autobiographical memory retrieval. We exposed participants to the stress *before* fully recollecting autobiographical memories (i.e., providing a full detailed account of the experience). Another study found different results when participants were exposed to a stressor (or control condition) *after* recalling past experiences - acute stress impaired the reconsolidation of autobiographical memories^97^. We suspect that a reason for this discrepancy is that this study targets different mechanisms related to reconsolidation depending on when the stressor is experienced in the retrieval pipeline. As pointed out by de Quervain, *et al*.^57^, glucocorticoids administered post-retrieval have a temporary effect on delayed recall, indicating that stress at this time may affect memory extinction processes, which are subject to spontaneous recovery, rather than reconsolidation. The findings from Schwabe and Wolf^97^ may reflect extinction processes rather than or in addition to the effects of reconsolidation. Testing this hypothesis is another important line of further research.

## Conclusion

In conclusion, our results provide new insights for how stress as a retrieval characteristic can alter specific aspects (i.e., stages) of recovering already consolidated autobiographical memories. Theoretically, these findings add an important piece to the puzzle of how memory mechanisms are affected by stress. Practically, these results have potential clinical implications for individuals with post-traumatic stress disorder or depression, who are affected by recalling traumatic or emotional memories. Our findings shed light on how traumatic memories can be strengthened (i.e., when they are retrieved in stressful contexts) and thus suggest that efforts should be made to prevent individuals from these groups from retrieving emotionally disturbing memories in stressful environments .
